# Global trends and emerging hotspots of macrophage research in kidney transplantation: a bibliometric and visualization analysis

**DOI:** 10.3389/fimmu.2026.1794759

**Published:** 2026-03-12

**Authors:** Tao Cai, Xiuli He, Shulin Cheng

**Affiliations:** 1 Department of Urology, the Affiliated Hospital of North Sichuan Medical College, Nanchong, Sichuan, China; 2 Department of Ultrasonography, the Affiliated Hospital of North Sichuan Medical College, Nanchong, Sichuan, China

**Keywords:** bibliometric analysis, fibrosis, ischemia – reperfusion injury, kidney transplantation, macrophages

## Abstract

**Background:**

Macrophages are plastic innate immune cells that couple inflammatory signaling with microvascular injury and alloimmune responses, thereby shaping rejection phenotypes and chronic allograft dysfunction after kidney transplantation. Yet, the mechanistic knowledge structure and emerging hotspots of this field remain insufficiently synthesized.

**Methods:**

Publications on kidney transplantation and macrophage-related research (2000–2025) were retrieved from the Web of Science Core Collection (n=538) and PubMed (n=385). Bibliometric and visualization analyses were performed using Microsoft Excel 2021, VOSviewer, CiteSpace, Charticulator, and Scimago Graphica, including trend analysis, collaboration mapping, journal and reference impact assessment, keyword co-occurrence and clustering, and burst detection. PubMed keyword analysis was used to validate and complement the WoSCC-based findings.

**Results:**

A total of 538 publications were identified, showing a steady increase over time. The United States and China were the major contributors, with extensive international collaboration networks. Co-citation and keyword analyses revealed that the research focus has evolved from early studies on graft injury and immune mechanisms to more recent hotspots such as immune infiltration, polarization, ferroptosis, transcriptomics, and machine learning. The timeline analysis further indicated a shift from foundational immunological mechanisms to mechanistic expansion and, more recently, to technology-driven and clinically oriented research, highlighting increasing emphasis on diagnosis, precision monitoring, and translational applications in kidney transplantation.

**Conclusion:**

Macrophage-focused kidney transplantation research is moving toward cell-state–resolved and technology-driven mechanistic frameworks that connect rejection pathology to long-term graft outcomes, providing a roadmap for biomarker development and targeted immunomodulation.

## 1 Introduction

Kidney transplantation remains the most effective treatment for end-stage renal disease (1). However, both early and late graft outcomes continue to be constrained by immune- and injury-driven complications, including ischemia–reperfusion injury (IRI), acute rejection, antibody-mediated rejection (ABMR), and progressive fibrotic remodeling that culminates in chronic allograft dysfunction (2, 3). Within this continuum, macrophages represent a central innate immune compartment capable of sensing tissue injury, orchestrating leukocyte recruitment, shaping antigen presentation, and amplifying endothelial and parenchymal damage (4, 5). The functional plasticity of macrophages-together with their crosstalk with dendritic cells, T cells, and endothelial cells-places them at the intersection of mechanistic immunology and clinically relevant transplant pathology, making them both key effectors and potential therapeutic/biomarker targets (6).

In parallel, diagnostic and classification frameworks have increasingly emphasized inflammatory lesions that are tightly linked to macrophage biology (7, 8). In particular, the Banff consensus has highlighted microvascular inflammation as a core histopathological basis for ABMR diagnosis, providing a widely adopted clinicopathological anchor for subsequent mechanistic studies on macrophage-mediated allograft injury (9, 10). Recent years have further witnessed a methodological shift toward high-dimensional and computational approaches, with signals of cross-disciplinary knowledge flow from molecular biology and genetics toward immunology and clinical medicine.

Notably, macrophage research in kidney transplantation is inherently interdisciplinary and large-scale, spanning transplant immunology, vascular pathology, fibrosis biology, molecular omics, and emerging computational analytics. The rapid expansion of publications across multiple scientific domains has generated a complex and fragmented knowledge landscape that is difficult to synthesize through traditional narrative reviews alone. Conventional reviews are typically hypothesis-driven and focus on selected mechanistic pathways, which may overlook latent knowledge structures, interdisciplinary linkages, and temporal research evolution within a large corpus of literature (11, 12). Bibliometric analysis provides a systematic approach to map scientific production and reveal research frontiers through co-authorship, co-occurrence, co-citation, and burst-detection analyses (13, 14). In this study, we retrieved publications on kidney transplantation and macrophage-related research from the Web of Science Core Collection (WoSCC) and performed comprehensive bibliometric analyses using tools including VOSviewer and CiteSpace. To enhance robustness, PubMed keyword analyses were conducted as an external validation and complementary perspective.

## 2 Materials and methods

This study retrieved publications on kidney transplantation and macrophage-related research from the Web of Science Core Collection (WoSCC) and PubMed. After screening, records were exported and analyzed using bibliometric tools, including CiteSpace. WoSCC data were used to characterize the overall research landscape and to perform analyses of countries/regions, authors, institutions, journals, keywords, and cited references, thereby delineating the field’s historical evolution, current status, and emerging trends. PubMed data were subsequently subjected to keyword analysis to validate and complement the WoSCC-based findings.

### 2.1 Data acquisition

On December 19, 2025, we first searched the Web of Science Core Collection (WoSCC) using the following query: TS=(“Kidney Transplantation” OR “Renal Transplantation” OR “Renal Transplantations” OR “Transplantations, Renal” OR “Transplantation, Kidney” OR “Kidney Transplantations” OR “Transplantations, Kidney” OR “Transplantation, Renal” OR “Grafting, Kidney” OR “Kidney Grafting”) AND TS=(“Macrophages” OR “Macrophage”). A total of 590 records published between 2000 and 2025 were retrieved. Only English-language articles and reviews were included, while 52 records of other document types were excluded, including Retracted Publication (n=1), Publication With Expression of Concern (n=1), Letter (n=1), Editorial Material (n=2), Early Access (n=2), Meeting Abstract (n=14), Proceeding Paper (n=27), and Non-English (n=4). Ultimately, 538 records were retained, comprising 469 articles and 69 reviews.

**Figure 1:**
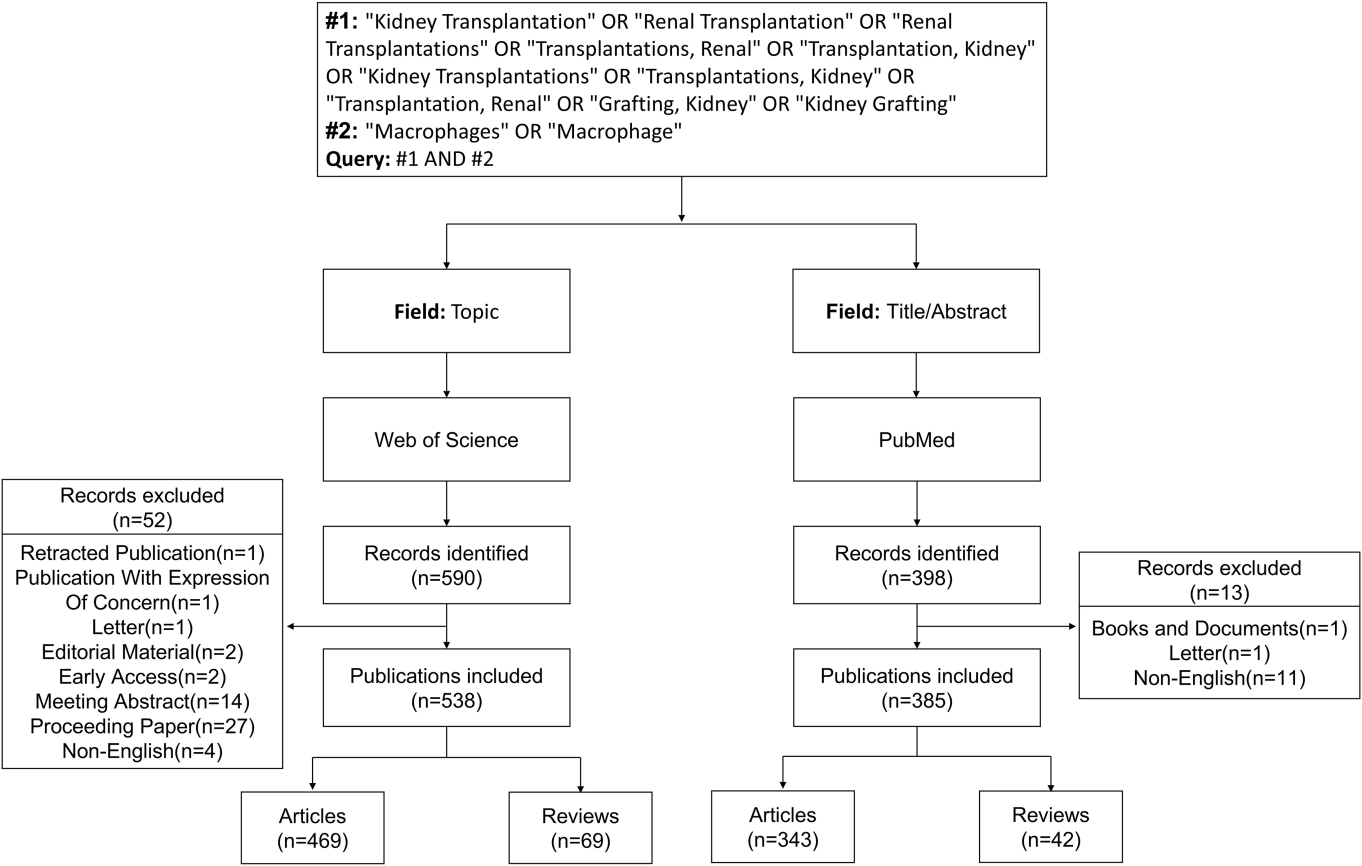
Flowchart of data retrieval and processing. Search strategies in WoSCC and PubMed, inclusion/exclusion criteria, record screening, and final datasets used for bibliometric analyses.

We then searched PubMed using the following query: (Kidney Transplantation [Title/Abstract] OR Renal Transplantation [Title/Abstract] OR Renal Transplantations [Title/Abstract] OR Transplantations, Renal [Title/Abstract] OR Transplantation, Kidney [Title/Abstract] OR Kidney Transplantations [Title/Abstract] OR Transplantations, Kidney [Title/Abstract] OR Transplantation, Renal [Title/Abstract] OR Grafting, Kidney [Title/Abstract] OR Kidney Grafting [Title/Abstract]) AND (Macrophages [Title/Abstract] OR Macrophage [Title/Abstract]). This search yielded 398 records published between 2000 and 2025. Only English-language articles and reviews were included, and 13 records of other document types were excluded, including Books and Documents (n=1), Letter (n=1), and Non-English (n=11). The g-index is the default node selection algorithm in CiteSpace, which adaptively selects nodes within each time slice based on their ranking, with the scaling factor k controlling the number of extracted items. In this study, k=20 was set for the PubMed dataset to ensure adequate keyword coverage while maintaining network clarity and comparability. Finally, 385 records were included, consisting of 343 articles and 42 reviews. **Figure 1** presents the detailed workflow of data acquisition and processing.

### 2.2 Bibliometric analysis and visualization

The processed data were subjected to bibliometric analysis and visualization using Microsoft Excel 2021, VOSviewer, CiteSpace, Charticulator, and Scimago Graphica.

VOSviewer (version 1.6.20) was employed to analyze and visualize country collaboration, author and institutional collaboration, as well as publishing and co-cited journals. VOSviewer (Visualization of Similarities Viewer), developed by Nees Jan van Eck and Ludo Waltman, is a software tool for constructing and visualizing bibliometric networks (15). Scimago Graphica was used to depict the annual publication outputs of countries with high publication volumes.

Charticulator was used to generate a chord diagram of international collaborations. Charticulator, a project of Microsoft Research, provides an online platform that enables researchers to customize visualizations without coding (https://donghaoren.org/charticulator/index.html). CiteSpace (version 6.4.R1) was applied to conduct dual-map overlay of journals, keyword co-occurrence analysis, co-citation analysis of references, and burst detection of keywords and references, with the results visualized accordingly. CiteSpace is a bibliometric and visualization software developed by Prof. Chaomei Chen’s team (16). The key CiteSpace parameters were explicitly defined as follows: the time span was set from 2000 to 2025, with a time slicing of 1 year per slice to capture fine-grained temporal evolution. The node selection was based on the g-index criterion to ensure balanced inclusion of influential nodes across time periods. For keyword analysis, the g-index scaling factor (k) was set to 5 for the Web of Science dataset and 10 for the PubMed dataset, considering the differences in database size and keyword density. In terms of network optimization, pruning strategies were applied to reduce noise and improve network clarity. Specifically, Pathfinder and Pruning the merged network were used for keyword co-occurrence analysis, whereas Pathfinder and Pruning sliced networks were employed for reference co-citation analysis. These parameter settings followed commonly recommended practices in bibliometric studies to ensure robustness, comparability, and reproducibility of the analytical results.

## 3 Results

### 3.1 Global trend in publication outputs and citations

**Figure 2:**
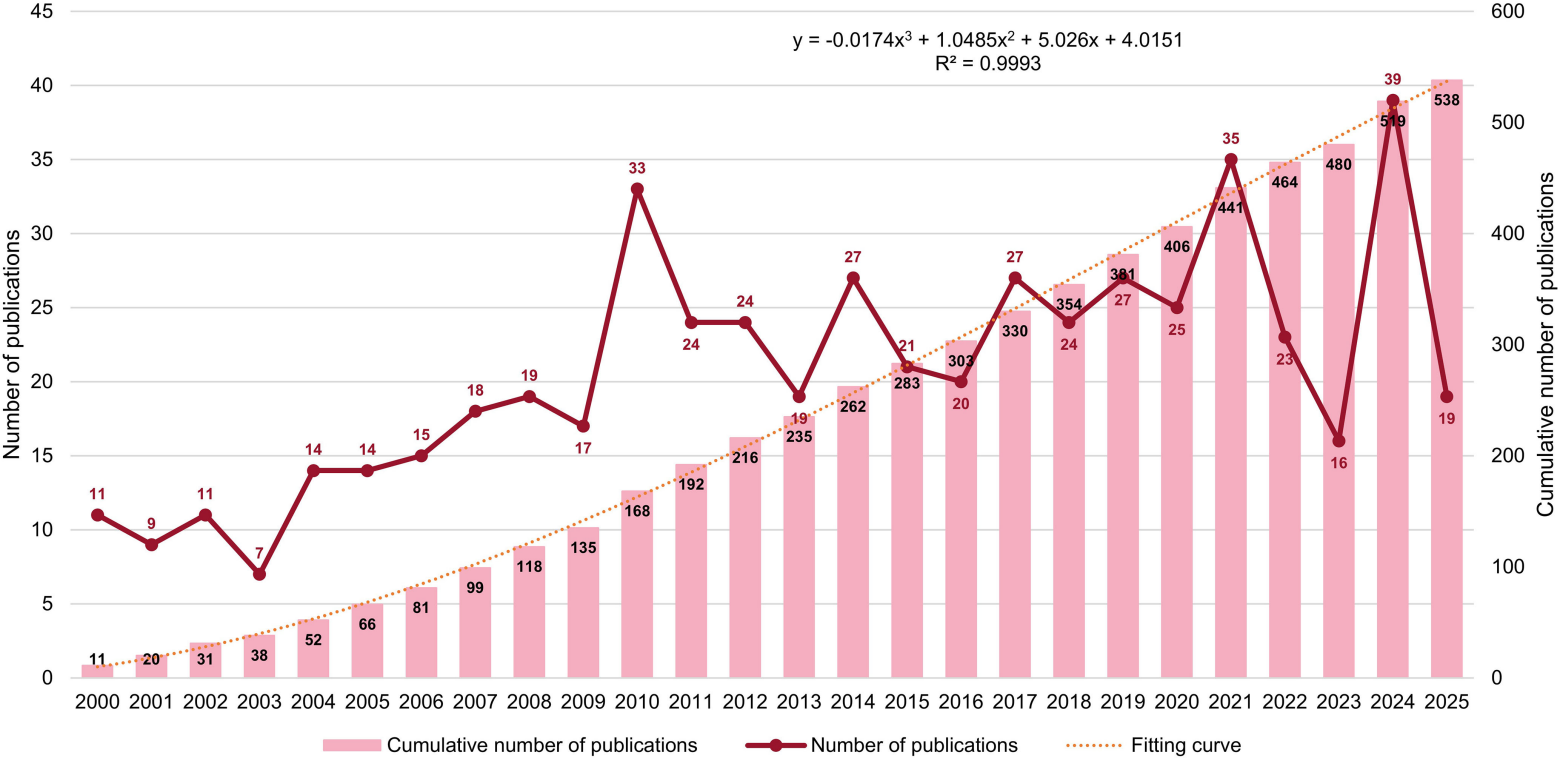
Annual publication trends in macrophage-related kidney transplantation research (2000–2025). Yearly outputs and cumulative publications reflecting the growth trajectory of the field.

Changes in publication output over time can reflect a field’s development and future direction. **Figure 2** shows the annual publications on macrophage-related research in kidney transplantation from 2000 to 2025, revealing an overall upward trend with fluctuations. From 2000 to 2009, the field was relatively small (14 papers/year) with slow growth. After 2010, publications increased markedly and remained higher (25 papers/year), with peaks in 2010 (33), 2021 (35), and 2024 (39), indicating sustained growth in attention over the past 15 years. Polynomial fitting of cumulative publications showed an excellent fit (R²=0.9993; red dashed line), suggesting that output will likely continue to rise.

The 538 included papers covered 49 research categories. **Table 1** lists the top 20 categories, led by Transplantation (203 papers), followed by Immunology (164), Surgery (148), Urology and Nephrology (108), and Cell Biology (39).

**Table 1:** Top 20 research categories by number of publications.

Rank	Web of science categories	Record count	Rank	Web of science categories	Record count
1	Transplantation	203	11	Physiology	13
2	Immunology	164	12	Medicine General Internal	12
3	Surgery	148	13	Chemistry Multidisciplinary	9
4	Urology Nephrology	108	14	Hematology	9
5	Cell Biology	39	15	Biology	8
6	Medicine Research Experimental	33	16	Endocrinology Metabolism	8
7	Biochemistry Molecular Biology	31	17	Genetics Heredity	7
8	Pharmacology Pharmacy	21	18	Medical Laboratory Technology	7
9	Multidisciplinary Sciences	19	19	Oncology	7
10	Pathology	13	20	Radiology Nuclear Medicine Medical Imaging	7

### 3.2 Distribution of countries/regions

A total of 51 countries/regions have contributed to this field. **Table 2** lists the top 10 countries by publication output. The United States ranked first in publications (number of publications, NP = 124), total citations (number of citations, NC = 4,826), and H-index (H = 40), confirming its leading role in both productivity and impact. Germany ranked second (NP = 113; NC = 4,188; H = 36), forming, together with the United States, the traditional leading group in this area. China ranked third (NP = 108), indicating strong output; however, its average citations per paper (average citations, AC = 17.97) and H-index (H = 24) were lower than those of the United States, Germany, and several European countries, suggesting that its international influence still needs improvement.

**Table 2:** Top 10 countries in terms of publication volume.

Rank	Country	NP	NC	AC	H-index
1	USA	124	4826	38.92	40
2	Germany	113	4188	37.06	36
3	China	108	1941	17.97	24
4	Netherlands	53	1378	26.00	23
5	United Kingdom	35	1545	44.14	22
6	Canada	35	1213	34.66	17
7	Japan	34	1028	30.24	18
8	France	29	1914	66.00	21
9	Australia	23	867	37.70	16
10	Italy	22	926	42.09	16

**Figure 3:**
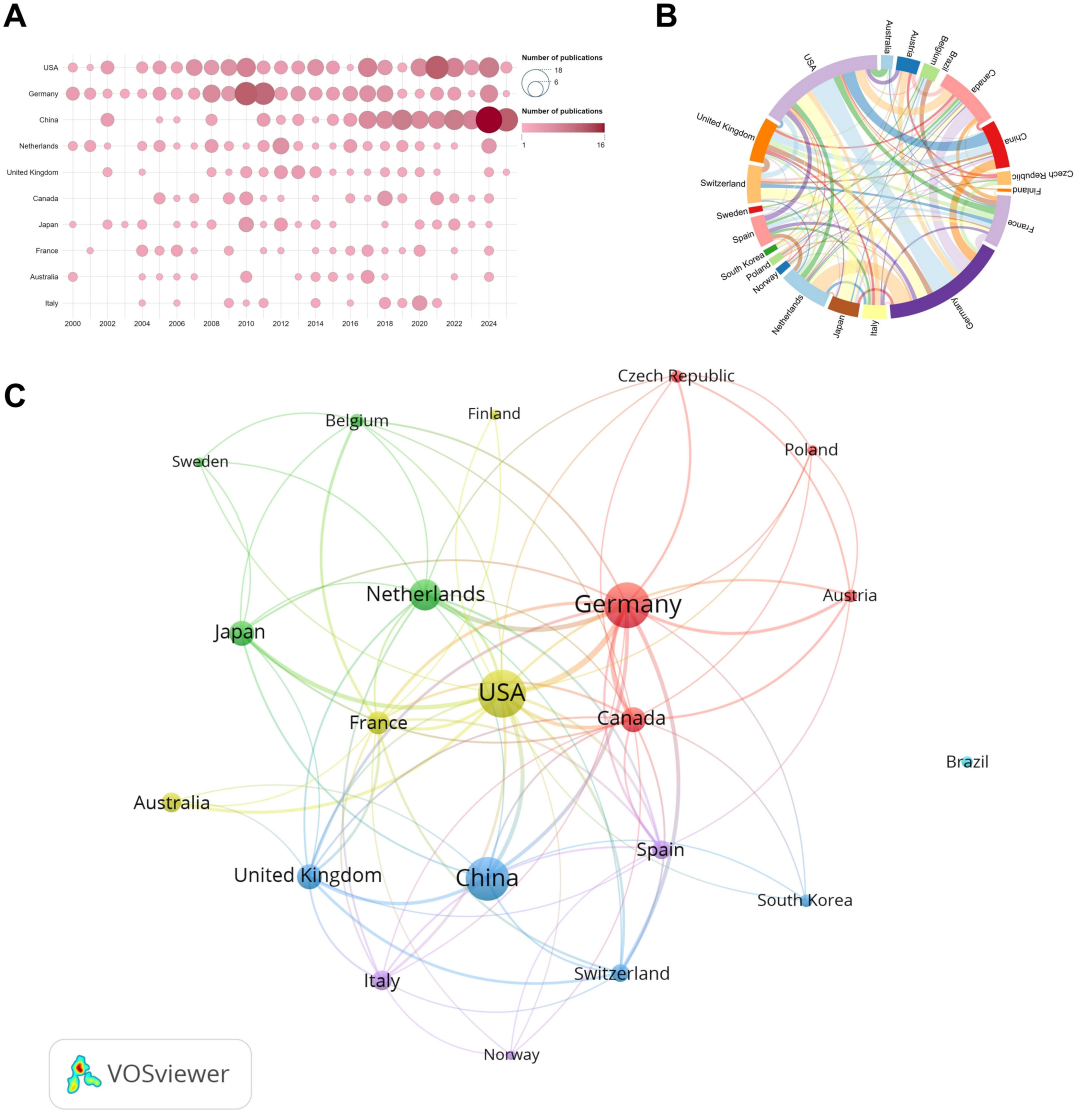
Country-level productivity and collaboration patterns. **(A)** Annual publication outputs of the top contributing countries. **(B)** International collaboration network among countries meeting the publication threshold; node size indicates total link strength (TLS) and link thickness indicates collaboration strength. **(C)** Chord diagram visualizing bilateral collaborations between countries, node size represents the frequency of occurrence, while link thickness indicates the strength of the relationship between nodes.

**Figure 3A** shows the annual publication trends of the top 10 countries. The first-tier countries, represented by the United States, maintained consistently high output over time, reinforcing their global leadership. In contrast, Germany exhibited a pronounced phase of peak productivity between 2008 and 2011, followed by a more stable pattern, suggesting concentrated efforts on key scientific questions or the formation of strong research teams during that period.

Country collaboration was analyzed in VOSviewer, including only countries with 5 more publications (n=21). **Figures 3B, C** present the collaboration network and chord diagram, where node size indicates total link strength (TLS). The network was centered on the United States and Germany (both TLS = 82), and their bilateral collaboration was the strongest (LS = 18). Germany collaborated extensively within Europe (e.g., the Netherlands, LS = 12; Switzerland, LS = 8), whereas the United States served as a global hub, linking with major countries including China (LS = 9) and Japan (LS = 10). Notably, despite China’s high output (NP = 107), its TLS was relatively low (TLS = 31), indicating that its international collaboration network remains less intensive and could be further strengthened.

### 3.3 Authors and institutions

**Figure 4:**
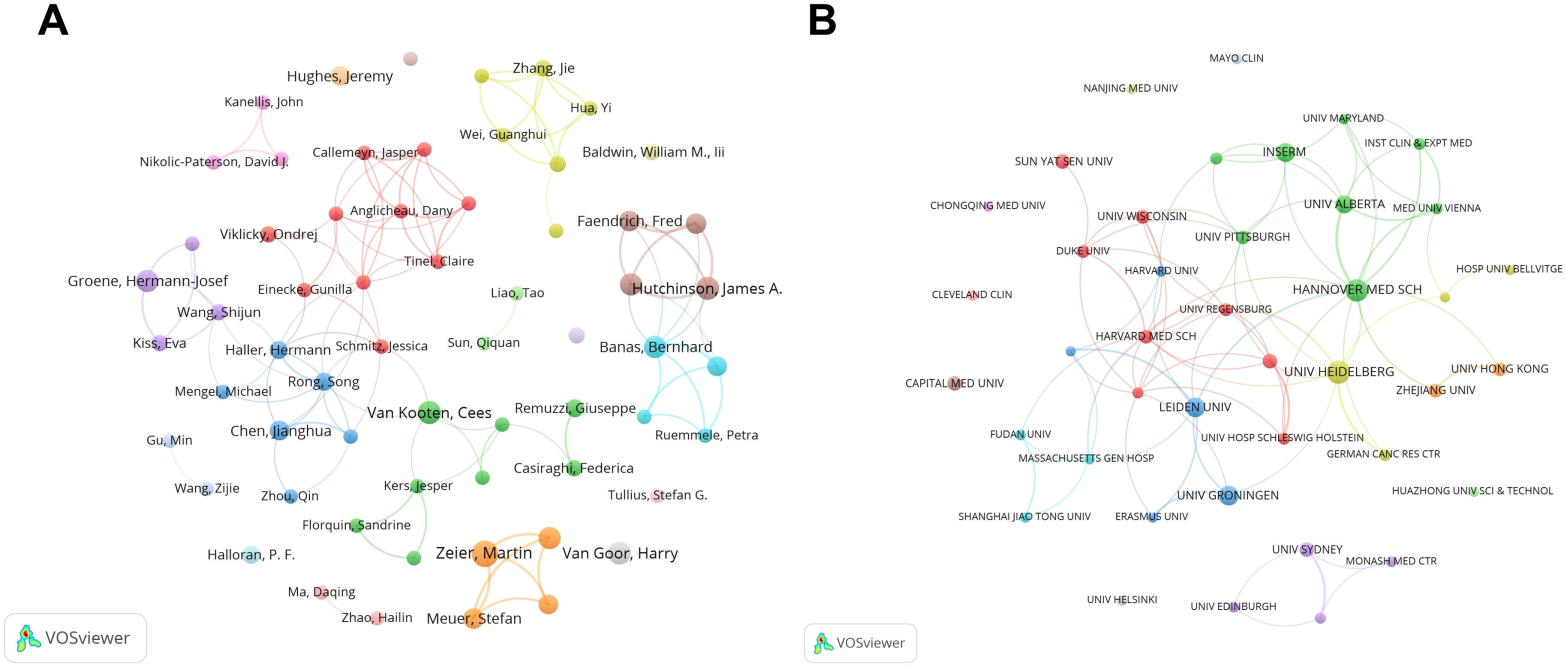
Collaboration networks of authors and institutions. **(A)** Author co-authorship network for authors meeting the minimum publication threshold. **(B)** Institutional collaboration network for institutions meeting the minimum publication threshold; node size reflects collaboration intensity (TLS) and links represent cooperative relationships.

A total of 3,627 authors contributed to this field. **Table 3** lists the top 10 authors by number of publications. Zeier, Martin ranked first with 13 publications, indicating sustained involvement; however, his total citations (NC = 208) and average citations per paper (AC = 16.00) were relatively modest. In contrast, Hutchinson, James A. and Geissler, Edward K. achieved markedly higher impact: with 10 and 9 publications, respectively, they accumulated 797 and 777 total citations, ranked among the top in average citations, and also showed high H-indices, highlighting their pioneering or central influence in the field. Using VOSviewer, we mapped authors with 4 more publications (n=62) to generate the author collaboration network (**Figure 4A**). The network comprised several tightly connected yet relatively independent collaboration clusters. A prominent core cluster was centered on Hutchinson, James A., Geissler, Edward K., and Riquelme, Paloma, who formed a strong triangular partnership (LS = 7–9). Together with collaborators such as Banas, Bernhard and Faendrich, Fred, they constituted a stable team with high total link strength (TLS = 22–28), representing a major research direction within the network.

**Table 3:** Top 10 authors by number of publications.

Rank	Author	NP	NC	AC	H-index
1	Zeier, Martin	13	208	16.00	10
2	Hutchinson, James A.	10	797	79.70	10
3	Van Goor, Harry	10	230	23.00	9
4	Van Kooten, Cees	10	172	17.20	7
5	Geissler, Edward K.	9	777	86.33	9
6	Banas, Bernhard	9	535	59.44	8
7	Groene, Hermann-Josef	9	478	53.11	8
8	Sommerer, Claudia	9	164	18.22	7
9	Riquelme, Paloma	8	448	56.00	8
10	Faendrich, Fred	8	430	53.75	7

825 institutions were involved in this research area. **Table 4** lists the top 10 institutions by publication output. The global institutional landscape was strongly led by key German institutions: UNIV HEIDELBERG ranked first in publications (26 papers), whereas HANNOVER MED SCH had the highest total citations (NC = 1,070), together forming dual centers of productivity and influence. In terms of academic impact, INSERM and UNIV HOSP REGENSBURG stood out with very high average citations per paper (approximately 61.35 and 71.50, respectively), indicating strong innovation and recognition. **Figure 4B** shows the institutional collaboration network for institutions with 5 more publications (n=41). The network was characterized by regional clustering and uneven collaboration intensity, with leading academic medical centers in Europe and North America acting as hubs. HANNOVER MED SCH was the most active hub, with the widest collaboration links (L = 12) and the highest TLS (TLS = 21), and it collaborated particularly closely with UNIV ALBERTA (LS = 6). HARVARD MED SCH (TLS = 12) formed a high-impact North America-UK collaboration axis through multiple connections with institutions such as DUKE UNIV and UNIV OXFORD. In contrast, several Chinese institutions met the publication threshold but showed relatively low TLS and sparse collaboration links.

**Table 4:** Top 10 institutions by publication volume.

Rank	Organization	NP	NC	AC	H-index
1	UNIV HEIDELBERG	26	683	26.27	16
2	HANNOVER MED SCH	24	1070	44.58	16
3	LEIDEN UNIV	19	435	22.89	11
4	UNIV GRONINGEN	18	540	30.00	14
5	INSERM	17	1043	61.35	12
6	UNIV ALBERTA	15	739	49.27	12
7	UNIV HOSP REGENSBURG	10	715	71.50	10
8	UNIV SYDNEY	10	342	34.20	10
9	CAPITAL MED UNIV	10	167	16.70	7
10	SUN YAT SEN UNIV	10	317	31.70	9

### 3.4 Journals

**Table 5** (left) lists the top 10 journals by number of publications. Transplantation (USA; IF = 5.1) published the most papers (n=58), followed by American Journal of Transplantation (Denmark; IF = 8.2; n=42), Kidney International (USA; IF = 12.6; n=28), and Frontiers in Immunology (Switzerland; IF = 5.9; n=28), indicating that these journals are favored publication venues in this field. **Table 5** (right) presents the top 10 most-cited journals. Transplantation (USA; IF = 5.1) ranked first with 2,107 citations, followed by American Journal of Transplantation (Denmark; IF = 8.2) and Kidney International (USA; IF = 12.6), with 1,785 and 1,300 citations, respectively, suggesting their substantial influence in this research area. **Figure 5A** shows the journal co-citation (coupling) network. The nodes represent journals with at least five publications (n=19); node size indicates publication output, and links indicate that two journals co-cited the same reference. **Figure 5B** overlays journal impact factors onto the co-citation network using a color gradient, where blue denotes lower impact factors and red denotes higher impact factors.

**Table 5:** The top 10 publishing journals by number of publications and the top 10 cited journals by citation frequency.

Rank	Journals	NP	Country	IF	Cited journals	NC	Country	IF
1	TRANSPLANTATION	58	USA	5.1	TRANSPLANTATION	2107	USA	5.1
2	AMERICAN JOURNAL OF TRANSPLANTATION	42	Denmark	8.2	AM J TRANSPLANT	1785	Denmark	8.2
3	KIDNEY INTERNATIONAL	28	USA	12.6	KIDNEY INT	1300	USA	12.6
4	FRONTIERS IN IMMUNOLOGY	28	Switzerland	5.9	J AM SOC NEPHROL	963	USA	9.4
5	TRANSPLANT INTERNATIONAL	26	Denmark	3	J IMMUNOL	866	USA	3.4
6	TRANSPLANT IMMUNOLOGY	24	Netherlands	1.4	J CLIN INVEST	509	USA	13.6
7	JOURNAL OF THE AMERICAN SOCIETY OF NEPHROLOGY	17	USA	9.4	NEPHROL DIAL TRANSPL	413	UK	5.6
8	NEPHROLOGY DIALYSIS TRANSPLANTATION	14	UK	5.6	FRONT IMMUNOL	390	Switzerland	5.9
9	PLOS ONE	10	USA	2.6	NEW ENGL J MED	363	USA	78.5
10	TRANSPLANTATION PROCEEDINGS	8	USA	0.8	P NATL ACAD SCI USA	335	USA	9.1

**Figure 5:**
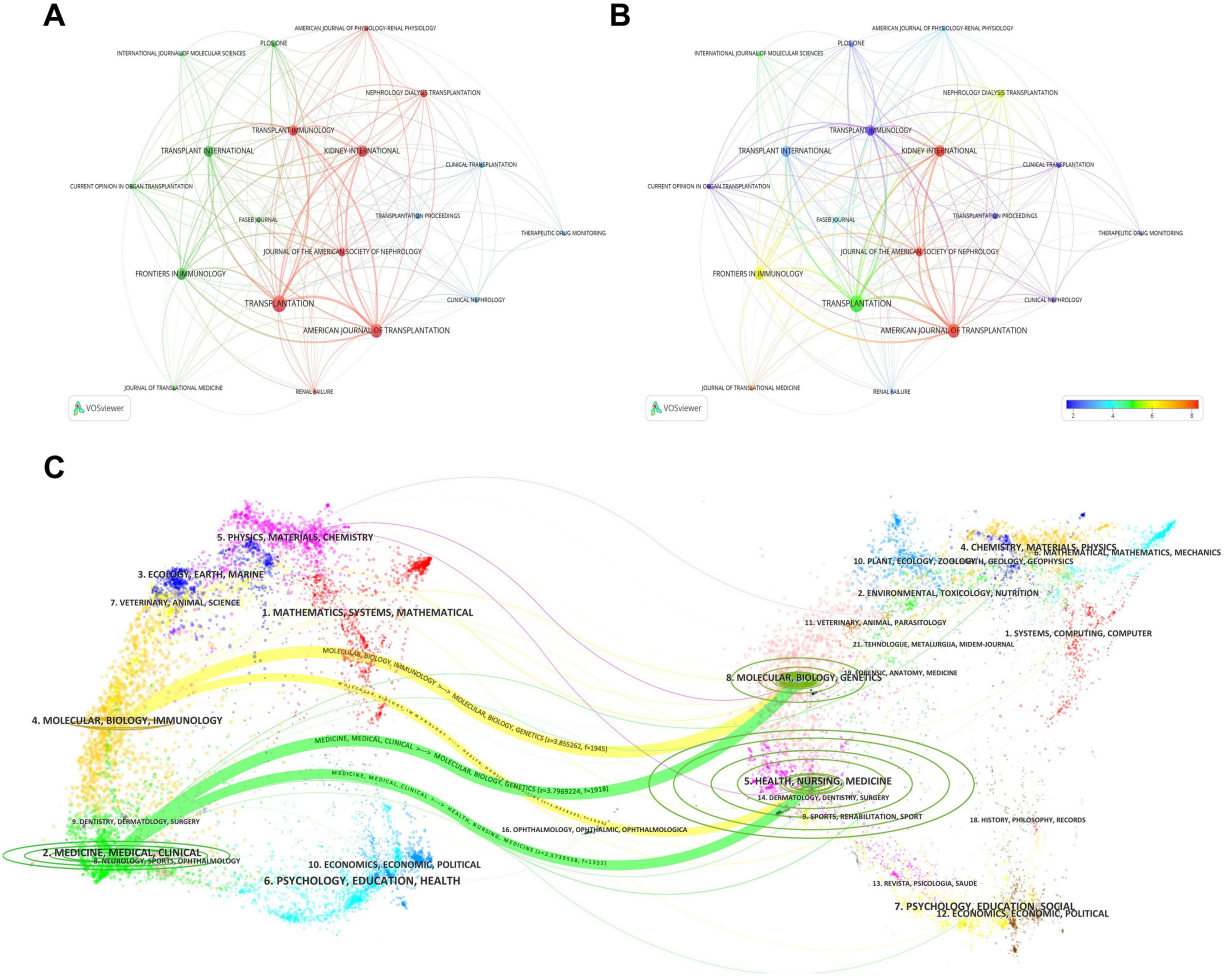
Journal landscape and knowledge flow. **(A)** Journal network based on coupling/co-citation relationships among journals meeting the publication threshold; node size indicates publication output and links represent shared citations. **(B)** Overlay visualization of journal impact factors on the journal network. **(C)** Dual-map overlay of source journals (citing; left) and cited journals (right), with colored trajectories indicating major citation pathways.

**Figure 5C** presents the dual-map overlay of source journals and cited journals for the 538 included records. Citing journals (left) represent application domains, whereas cited journals (right) reflect the disciplinary knowledge base. The colored curved lines indicate citation pathways, illustrating cross-field connections and the publishing-citing activities of each domain. Clustering based on the built-in Z-score algorithm identified four major knowledge-flow citation pathways, indicated by the colors of the cited regions; the width of each trajectory is proportional to the Z-score of citations. Overall, the dominant citation trajectories originate from “MOLECULAR, BIOLOGY, GENETICS” and “HEALTH, NURSING, MEDICINE”, and extend toward the frontier areas of “MOLECULAR, BIOLOGY, IMMUNOLOGY” and “MEDICINE, MEDICAL, CLINICAL.” Notably, the pathway from “MOLECULAR, BIOLOGY, GENETICS” to “MOLECULAR, BIOLOGY, IMMUNOLOGY” exhibited the highest Z value (z=3.855), underscoring its prominence and influence.

### 3.6 Keywords and hotspots

**Figure 6:**
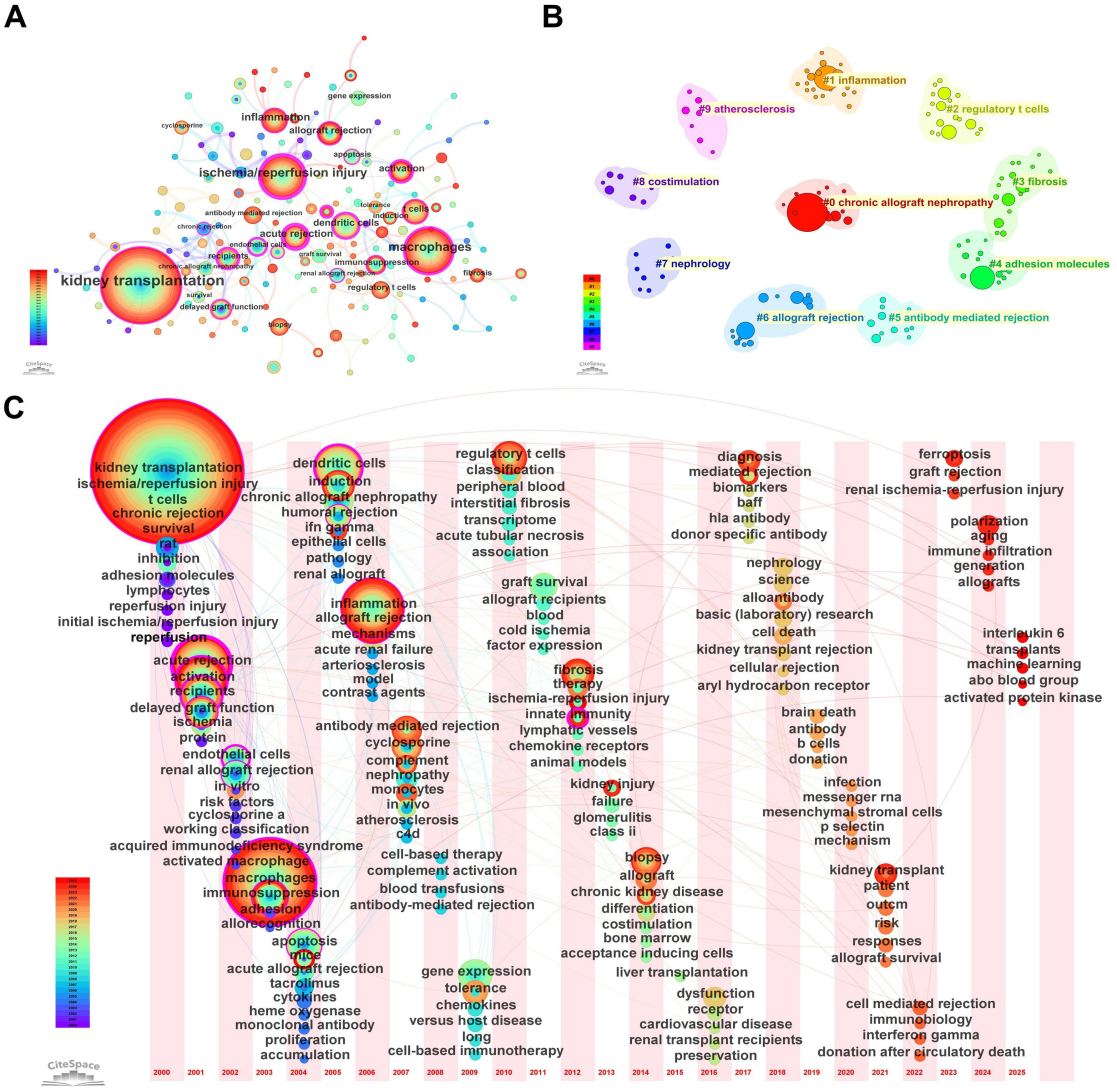
Keyword mapping and thematic evolution (WoSCC). **(A)** Keyword co-occurrence network; node size represents keyword frequency and links indicate co-occurrence relationships. **(B)** Keyword clustering map generated by CiteSpace (LLR algorithm). **(C)** Keyword timeline (time-zone) visualization showing the temporal distribution and evolution of major themes.

**Figure 6A** shows the keyword co-occurrence network, where nodes represent keywords and node size reflects keyword frequency (larger nodes indicate higher occurrence). Overall, research in this field is organized around “kidney transplantation” (359 occurrences) as the central topic, with “ischemia/reperfusion injury” exhibiting the highest betweenness centrality (0.53), indicating its pivotal bridging role in the network. Although “macrophages” (144 occurrences) is highly frequent, its centrality (0.28) suggests it functions primarily as a core research object rather than the main conceptual connector. In contrast, keywords such as “acute rejection” (centrality 0.44), “dendritic cells” (0.29), “endothelial cells” (0.29), and “innate immunity” (0.31), despite lower frequencies, show strong bridging roles, implying that macrophage-related studies are deeply embedded in interconnected pathophysiological modules including acute rejection, antigen presentation, endothelial injury, and innate immune activation. In addition, terms such as “inflammation” (66 occurrences), “apoptosis” (centrality 0.19), and “fibrosis” (18 occurrences) outline a disease-progression continuum from early injury and inflammation to chronic fibrotic remodeling. The presence of clinically oriented terms such as “delayed graft function,” “biopsy,” and “biomarkers” further suggests an increasing translation of mechanistic findings into clinical diagnosis and prognostic assessment. Collectively, the network indicates that macrophage research in kidney transplantation represents an interdisciplinary hotspot linking fundamental immune mechanisms with key clinical transplant challenges. **Figure 6B** presents keyword clustering based on CiteSpace using the log-likelihood ratio (LLR) algorithm to extract cluster labels from the keyword field. Ten clusters were identified, with Q = 0.7125 (>0.3) indicating a significant clustering structure and S = 0.8968 (>0.7) indicating high reliability. The ten clusters were: #0 chronic allograft nephropathy, #1 inflammation, #2 regulatory t cells, #3 fibrosis, #4 adhesion molecules, #5 antibody mediated rejection, #6 allograft rejection, #7 nephrology, #8 costimulation, and #9 atherosclerosis, representing major research themes in this field.

Based on the keyword timeline map (**Figure 6C**) and the top 16 burst keywords in **Table 6**, the evolution of this field can be broadly divided into three stages. Stage I (2000–2005): Burst keywords formed the classical foundational framework of the field, centered on “kidney transplantation,” “ischemia/reperfusion injury,” “acute/chronic rejection,” and “t cells,” focusing on transplantation procedures, early ischemia–reperfusion injury, and T-cell–driven acute rejection. Stage II (2006–2015): The research perspective shifted from generalized “rejection” to more refined subtypes and mechanisms. The emergence of “antibody mediated rejection,” “humoral rejection,” and the diagnostic marker “c4d” reflects the establishment of ABMR as a distinct and important research direction. Meanwhile, rising interest in “fibrosis,” “monocytes,” and “chronic kidney disease” indicates a transition from acute events toward long-term graft outcomes and chronic injury mechanisms. Stage III (2016–present, especially after 2020): Keywords became increasingly frontier- and technology-driven. Mechanistic attention expanded to emerging topics such as “ferroptosis,” “polarization,” “senescence,” and “immune infiltration.” Methodological innovations were reflected by terms including “transcriptome,” “machine learning,” and “donation after circulatory death,” highlighting the growing roles of omics technologies, artificial intelligence, and evolving clinical practice in driving new research momentum. Meanwhile, the burst of clinically oriented keywords such as “kidney transplant” (2021–2025) and “diagnosis” (2022–2023) strongly points toward translational practice, indicating a shift toward converting novel mechanisms into more precise diagnostic tools and optimized transplantation strategies.

**Table 6:** Keyword emergence related to kidney transplantation and macrophage research.

Keywords	Year	Strength	Begin	End	2000 - 2025
chronic rejection	2000	4.92	2000	2008	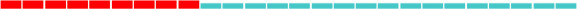
rat	2000	4.47	2000	2006	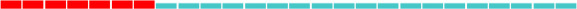
apoptosis	2004	3.58	2004	2014	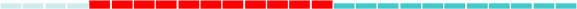
chronic allograft nephropathy	2005	3.63	2005	2010	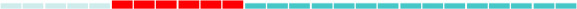
nephropathy	2007	3.68	2007	2010	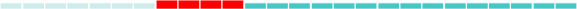
endothelial cells	2002	3.52	2007	2009	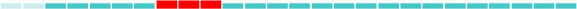
gene expression	2009	6.94	2009	2015	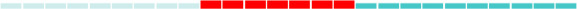
dendritic cells	2005	5.04	2010	2015	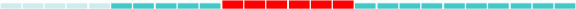
graft survival	2011	5.13	2011	2015	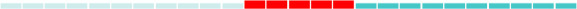
t cells	2000	4.67	2014	2021	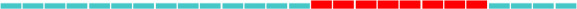
dysfunction	2016	3.83	2016	2018	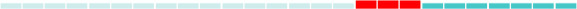
fibrosis	2012	3.34	2016	2017	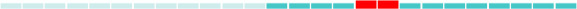
tolerance	2009	3.32	2019	2020	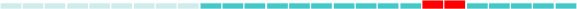
antibody mediated rejection	2007	4.43	2020	2023	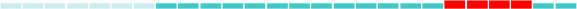
kidney transplant	2021	3.99	2021	2025	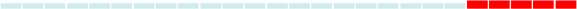
diagnosis	2017	3.54	2022	2023	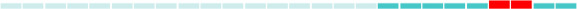

Each timeline is divided into 26 segments representing the years from 2000 to 2025. Three colors are used: light blue indicates the full time span, dark blue marks the period after the keyword first appeared, and red highlights the years during which the keyword exhibited a burst.

### 3.7 Comparative analysis of keywords in the PubMed

**Figure 7:**
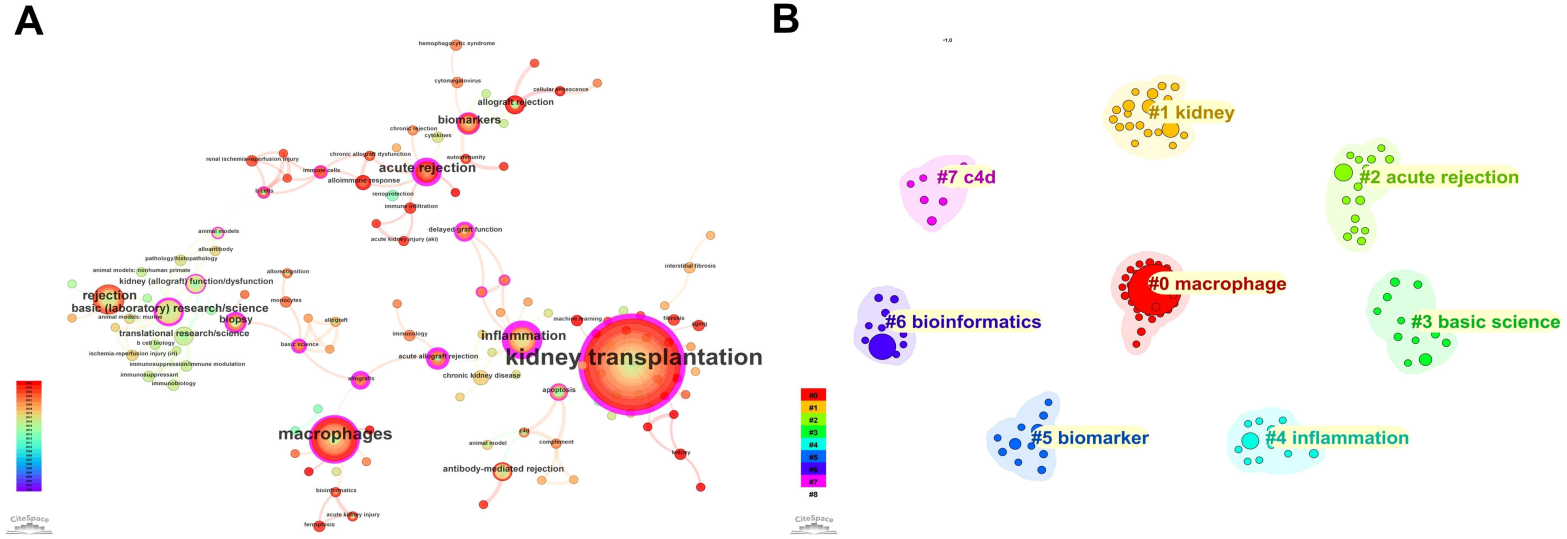
Keyword validation and refinement using PubMed. **(A)** Keyword co-occurrence network derived from the PubMed dataset. **(B)** Keyword clustering map (LLR algorithm) based on PubMed records, used to validate and complement WoSCC-derived themes.

Using the PubMed database, 385 records were retrieved. Based on the g-index (k=20), keyword extraction was performed on the included 385 articles, yielding 151 keywords. **Figure 7A** presents the keyword co-occurrence network. Keyword analysis based on the PubMed dataset robustly validates and further enriches the conclusions drawn from Web of Science, while also revealing more nuanced research tendencies. Both databases consistently identify “kidney transplantation” and “inflammation” as indispensable core nodes. Notably, “inflammation” shows exceptionally high betweenness centrality in PubMed (0.85), further reinforcing its role as a key hub linking diverse pathological processes. The continued prominence of keywords such as “acute rejection” and “macrophages” also supports the consistent positioning of acute rejection and macrophages as central research foci. Importantly, PubMed highlights a more fine-grained basic and translational research orientation. On one hand, “basic (laboratory) research/science” emerges as an independent keyword with high centrality, underscoring the intrinsic value of fundamental scientific exploration. On the other hand, numerous terms related to specific molecular markers, cell subtypes, emerging cell-death modalities, and computational approaches-such as “ccl2,” “cd163+ m2 macrophage,” “ferroptosis,” “bioinformatics,” and “machine learning”-are more prominent than in WoS. This indicates that current research is rapidly moving toward deeper mechanistic dissection and interdisciplinary methodological integration. These PubMed-derived findings therefore complement and strengthen the Stage III characterization of “frontier exploration and technology convergence,” highlighting increasing complexity at both the molecular and methodological levels.

**Figure 7B** shows keyword clustering using CiteSpace with the log-likelihood ratio (LLR) algorithm to extract cluster labels from the keyword field. Eight clusters were generated: #0 macrophage, #1 kidney, #2 acute rejection, #3 basic science, #4 inflammation, #5 biomarker, #6 bioinformatics, and #7 c4d. Comparing the clustering structures from PubMed and Web of Science further validates and refines the evolution of the field’s knowledge system. In contrast to the more parallel, pathology-oriented thematic clusters in WoS, PubMed displays a clearer vertical integration across basic–clinical–technical dimensions. Specifically, PubMed cluster #3 directly aggregates “basic research,” “translational research,” together with “animal models” and “pathology,” strongly corroborating the translational trend observed in WoS and establishing it as a central research paradigm. Meanwhile, clusters #0, #2, #4, and #5 concentrate on topics such as acute rejection and B-cell-related mechanisms, diagnosis and biopsy, inflammation and macrophage polarization, and biomarkers and senescence. These themes align closely with the corresponding WoS clusters, while PubMed further reveals finer-resolution analyses of immune subsets and molecular mechanisms.

### 3.8 Citation and co-citation analysis

**Figure 8:**
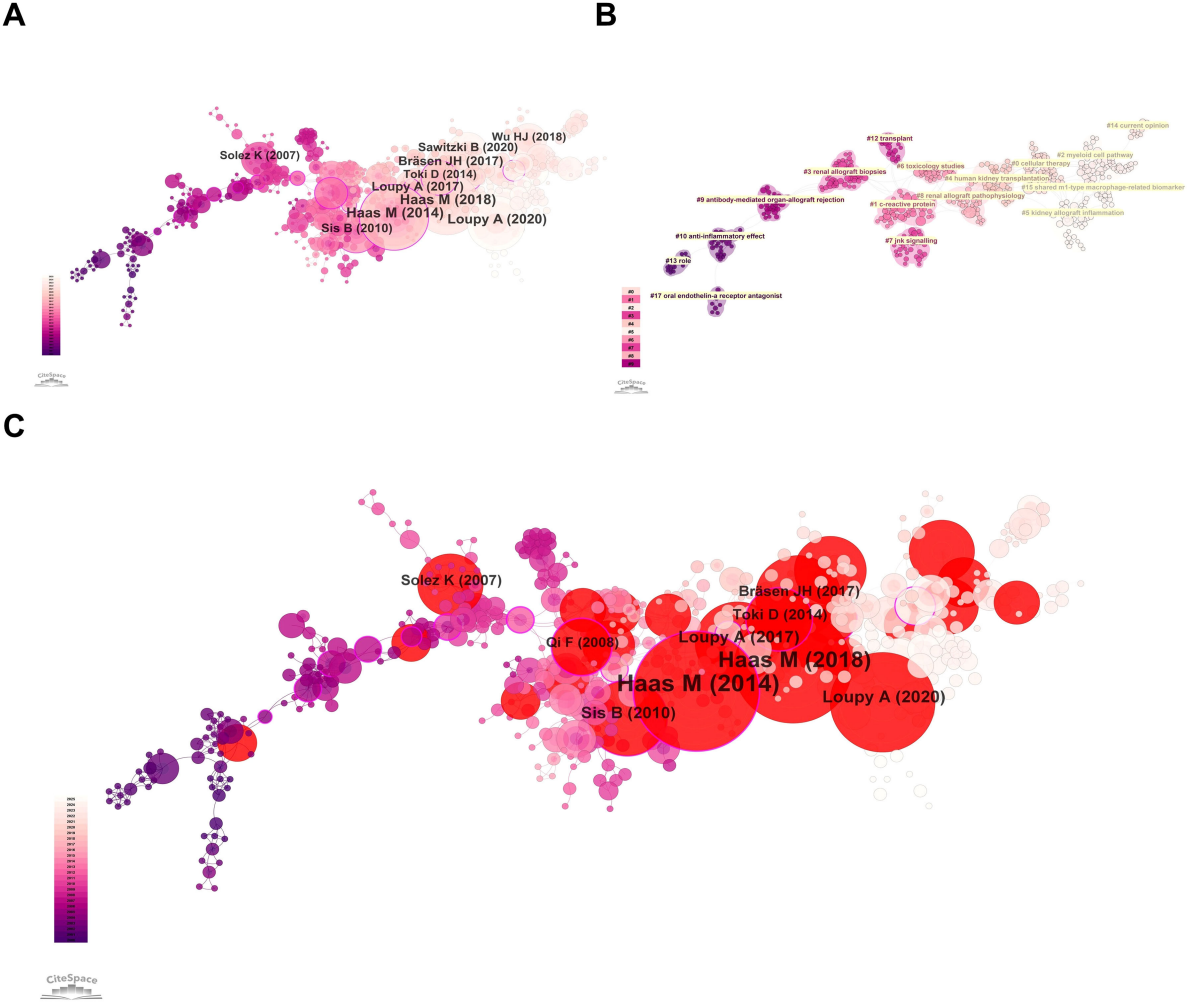
Knowledge base and emerging frontiers from reference analysis. **(A)** Reference co-citation network with labels for highly co-cited references. **(B)** Reference clustering network generated using the LLR algorithm. **(C)** Reference burst detection map highlighting references with strong citation bursts over time.

**Figure 8A** shows the reference co-citation network. Labels indicate the 10 most frequently co-cited references, including the first author and publication year. The largest node corresponds to the paper by Haas, M. et al. published in 2014 in the American Journal of Transplantation, entitled “Banff 2013 Meeting Report: Inclusion of C4d-Negative Antibody-Mediated Rejection and Antibody-Associated Arterial Lesions”. This reference received the highest citation frequency (19 citations), indicating its strongest influence and broad recognition in this field. A key reason is that it serves as a landmark consensus document in transplant pathology.

Specifically, the report established histological microvascular inflammation including glomerulitis and peritubular capillaritis-as central criteria for diagnosing antibody-mediated rejection (ABMR). Because macrophages are key effector cells driving such inflammatory lesions, most subsequent studies investigating macrophage roles in renal allograft rejection-particularly antibody-mediated injury-whether focusing on mechanisms, phenotypic polarization, or biomarker potential, cite this consensus to anchor the clinical and pathological context of their research questions.

On this basis, we further conducted clustering analysis of the co-cited references. Using the log-likelihood ratio (LLR) algorithm, cluster labels were extracted from the title field, yielding 16 major clusters from 888 references. The modularity was Q = 0.9114 (>0.3), indicating a significant clustering structure, and the mean silhouette was S = 0.961 (>0.7), indicating high reliability. The co-citation cluster network is shown in **Figures 8B**, including #0 cellular therapy, #1 c-reactive protein, #2 myeloid cell pathway, #3 renal allograft biopsies, #4 human kidney transplantation, #5 kidney allograft inflammation, #6 toxicology studies, #7 jnk signalling, #8 renal allograft pathophysiology, #9 antibody-mediated organ-allograft rejection, #10 anti-inflammatory effect, #12 transplant, #13 role, #14 current opinion, #15 shared m1-type macrophage-related biomarker, and #17 oral endothelin-a receptor antagonist. **Table 7** lists the top 25 references with the strongest citation bursts. **Figure 8C** highlights burst references (red nodes), with labels indicating those with higher burst strength. Notably, the strongest burst again corresponded to the 2014 American Journal of Transplantation article by Haas, M. et al.

**Table 7:** Bursting references related to kidney transplantation and macrophage research.

References	Year	Strength	Begin	End	2000 - 2025
Racusen LC, 1999, KIDNEY INT, V55, P713, DOI 10.1046/j.1523-1755.1999.00299.x, DOI	1999	3.82	2002	2004	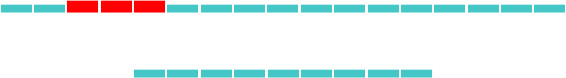
Nankivell Brian J, 2003, NEW ENGLAND JOURNAL OF MEDICINE, V349, P2326, DOI 10.1056/NEJMoa020009, DOI	2003	3.58	2005	2007	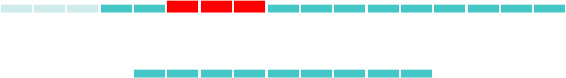
Solez K, 2007, AM J TRANSPLANT, V7, P518, DOI 10.1111/j.1600-6143.2006.01688.x, DOI	2007	5.65	2009	2011	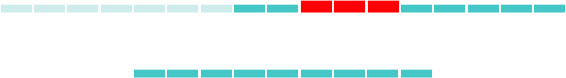
Solez K, 2008, AM J TRANSPLANT, V8, P753, DOI 10.1111/j.1600-6143.2008.02159.x, DOI	2008	4.52	2010	2012	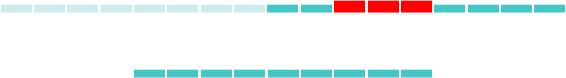
Ricardo SD, 2008, J CLIN INVEST, V118, P3522, DOI 10.1172/JCI36150, DOI	2008	3.66	2010	2013	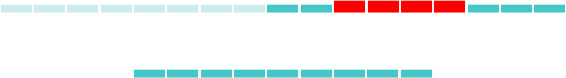
Sis B, 2010, AM J TRANSPLANT, V10, P464, DOI 10.1111/j.1600-6143.2009.02987.x, DOI	2010	6.28	2011	2015	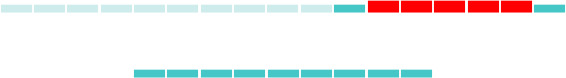
Qi F, 2008, TRANSPLANTATION, V86, P1267, DOI 10.1097/TP.0b013e318188d433, DOI	2008	5.38	2011	2013	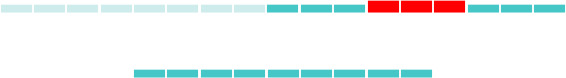
Hutchinson JA, 2011, J IMMUNOL, V187, P2072, DOI 10.4049/jimmunol.1100762, DOI	2011	3.86	2012	2015	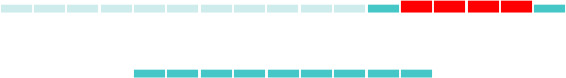
Haas M, 2014, AM J TRANSPLANT, V14, P272, DOI 10.1111/ajt.12590, DOI	2014	9.16	2014	2019	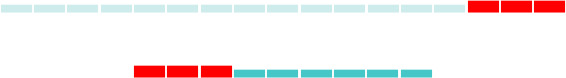
Riquelme P, 2013, MOL THER, V21, P409, DOI 10.1038/mt.2012.168, DOI	2013	3.62	2014	2018	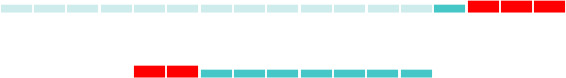
Huang G, 2014, AM J TRANSPLANT, V14, P1061, DOI 10.1111/ajt.12674, DOI	2014	3.94	2015	2018	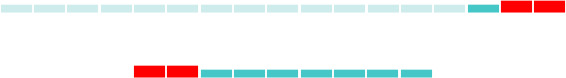
Toki D, 2014, AM J TRANSPLANT, V14, P2126, DOI 10.1111/ajt.12803, DOI	2014	5.78	2017	2019	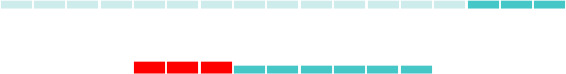
Loupy A, 2017, AM J TRANSPLANT, V17, P28, DOI 10.1111/ajt.14107, DOI	2017	6.38	2018	2021	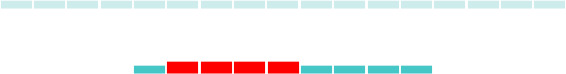
Bräsen JH, 2017, KIDNEY INT, V92, P0, DOI 10.1016/j.kint.2017.01.029, DOI	2017	5.6	2018	2022	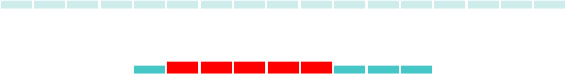
Bergler T, 2016, PLOS ONE, V11, P0, DOI 10.1371/journal.pone.0156900, DOI	2016	4.26	2018	2019	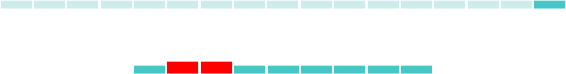
Haas M, 2018, AM J TRANSPLANT, V18, P293, DOI 10.1111/ajt.14625, DOI	2018	8.65	2019	2022	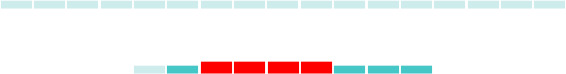
Wang YY, 2017, J AM SOC NEPHROL, V28, P2053, DOI 10.1681/ASN.2016050573, DOI	2017	3.81	2019	2022	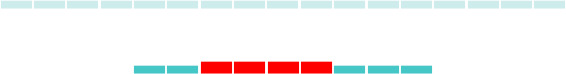
Wu HJ, 2018, J AM SOC NEPHROL, V29, P2069, DOI 10.1681/ASN.2018020125, DOI	2018	4.85	2020	2023	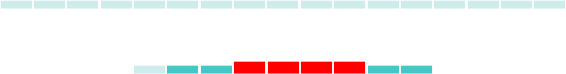
van den Bosch TPP, 2017, AM J TRANSPLANT, V17, P2659, DOI 10.1111/ajt.14280, DOI	2017	4.04	2020	2021	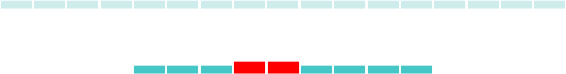
Loupy A, 2020, AM J TRANSPLANT, V20, P2318, DOI 10.1111/ajt.15898, DOI	2020	6.46	2021	2025	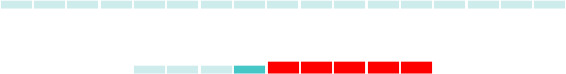
Sawitzki B, 2020, LANCET, V395, P1627, DOI 10.1016/S0140-6736(20)30167-7, DOI	2020	4.84	2021	2025	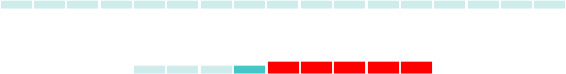
Malone AF, 2020, J AM SOC NEPHROL, V31, P1977, DOI 10.1681/ASN.2020030326, DOI	2020	4.24	2021	2023	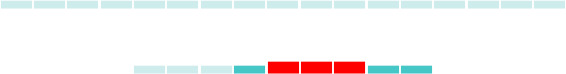
Dangi A, 2020, JCI INSIGHT, V5, P0, DOI 10.1172/jci.insight.141321, DOI	2020	3.61	2021	2025	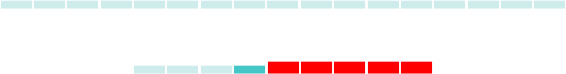
Dai HH, 2020, SCIENCE, V368, P1122, DOI 10.1126/science.aax4040, DOI	2020	3.6	2022	2025	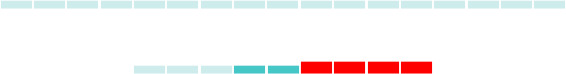
Nieuwenhuijs-Moeke GJ, 2020, J CLIN MED, V9, P0, DOI 10.3390/jcm9010253, DOI	2020	4.02	2023	2025	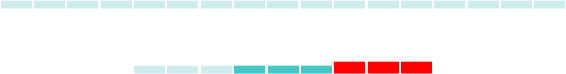

Each timeline is divided into 26 segments representing the years from 2000 to 2025. Three colors are used: light blue indicates the full time span, dark blue marks the period after the keyword first appeared, and red highlights the years during which the keyword exhibited a burst.

## 4 Discussion

This bibliometric and visualization analysis systematically mapped the intellectual landscape of macrophage-related research in kidney transplantation from 2000 to 2025 using WoSCC and PubMed. Beyond describing growth and collaboration, the network structure provides a mechanism-centered view of how the field conceptualizes graft injury: macrophages sit at the intersection of inflammatory amplification, microvascular pathology, rejection phenotypes, and long-term remodeling. Importantly, the two-database design not only confirmed stable “core” mechanisms but also revealed a clear shift toward cell-state–resolved and technology-enabled mechanistic dissection, suggesting that macrophage biology is increasingly used as a unifying framework to connect immunopathology with clinically actionable stratification. This keyword-driven analytical framework is consistent with recent bibliometric studies in immunology and nephrology, which emphasize that keyword co-occurrence and burst analysis can effectively reveal mechanistic evolution and emerging translational hotspots (17, 18).

Across both databases, “kidney transplantation” anchored the knowledge network, while “inflammation” emerged as a dominant connector-especially in PubMed where it showed exceptionally high centrality-indicating that inflammatory processes are the principal wiring that links otherwise distinct mechanistic modules. This topological feature is biologically plausible: macrophages translate tissue-derived danger cues and alloimmune signals into cytokine/chemokine programs, thereby shaping antigen-presenting cell activity, T-cell priming, endothelial activation, and parenchymal stress responses (19, 20). Notably, although “macrophages” ranked among the most frequent keywords, its centrality was lower than several bridge terms such as “acute rejection,” “innate immunity,” “endothelial cells,” and “dendritic cells,” implying that the field increasingly interprets macrophage effects through interaction networks rather than macrophages alone. From a mechanistic and pathway perspective, this inflammation-centered network aligns with growing evidence that oxidative stress–inflammation crosstalk and immune signaling pathways play central roles in renal injury and transplant-related immune remodeling (21, 22). In keyword-network terms, this suggests that macrophages function more as an integrative immunological node embedded within broader rejection and vascular injury modules rather than as an isolated research focus. In other words, macrophage research in transplantation has matured from a “cell-of-interest” narrative into a systems immunology narrative, where macrophages are pivotal because they couple innate activation to adaptive effector pathways and vascular injury, and because they shape the tissue repair/fibrosis trajectory.

Reference co-citation analysis highlighted the Banff 2013/2014 update by Haas et al. as the most influential and strongest-burst reference (23). This is mechanistically informative: Banff reframed ABMR diagnosis by emphasizing microvascular inflammation, which is a macrophage-rich lesion pattern. Consequently, studies examining macrophage infiltration, polarization markers, and macrophage-associated transcriptomic signatures naturally cite Banff to define the clinical-pathological ground truth of antibody-associated injury (24). From a mechanistic standpoint, this dominance indicates that macrophage biology in renal allografts is being interpreted through the lens of endothelial–microvascular injury and antibody-associated inflammation, rather than only through classical T-cell–mediated paradigms (25). The prominence of clusters related to “antibody-mediated organ-allograft rejection,” “kidney allograft inflammation,” and “renal allograft biopsies” further supports that the central battlefield of macrophage mechanisms is increasingly the microvasculature and the biopsy-defined inflammatory architecture (26, 27).

A key evolution over the past decade is the shift from more macrophages equals worse outcomes toward functional heterogeneity and state transitions. The emergence of polarization-related terms and specific markerssuggests a growing effort to distinguish inflammatory macrophage programs that amplify rejection from reparative programs that may either resolve inflammation or, paradoxically, promote fibrosis (28, 29). This duality likely explains why “fibrosis” and chronic allograft dysfunction clusters remain persistent: macrophage-derived mediators can simultaneously dampen acute inflammation and drive extracellular matrix remodeling, depending on the local milieu, antigenic stimulation, and metabolic constraints (30, 31). In practical terms, this maturation supports a biomarker strategy that is less focused on total macrophage density and more on cell-state signatures-either immunohistochemical panels or transcriptomic modules-that can stratify patients by active microvascular inflammation, chronic remodeling risk, or therapeutic responsiveness (32, 33).

The post-2016, especially post-2020, burst of terms such as “ferroptosis,” “senescence,” and “immune infiltration” indicates that the macrophage field is expanding into non-classical injury biology and tissue ecology. These topics reflect new mechanistic “interaction surfaces” between macrophages and graft parenchyma: ferroptotic stress and senescent signaling can reprogram macrophage activation; conversely, macrophage cytokines and reactive species can promote regulated cell death pathways and persistent senescence-associated inflammation (34, 35). These emerging hotspots may directly address key clinical pain points in kidney transplantation, including the early detection of subclinical rejection, differentiation between acute and chronic injury, and prediction of long-term graft dysfunction. Immune infiltration profiling and transcriptomic signatures could support more sensitive diagnostic tools and complement traditional biopsy-based assessment, while machine learning–assisted models may enhance risk stratification and individualized prognosis evaluation. The bibliometric emergence of these terms suggests that transplantation research is converging with broader immunometabolism and cell-death biology, which may yield novel therapeutic leverage points. The recent burst of clinically oriented keywords such as “kidney transplant” and “diagnosis” further indicates a shift toward translational and practice-driven research, emphasizing the application of mechanistic insights to diagnostic optimization and clinical management. This translational orientation is consistent with recent nephrology research emphasizing targeted pathway modulation and integrative therapeutic strategies to improve clinical outcomes in kidney diseases (36, 37). Importantly, these frontier mechanisms also provide an explanatory bridge between early inflammatory lesions and later fibrotic remodeling, aligning with the observed chronic allograft nephropathy and fibrosis clusters (38, 39).

PubMed keywords disproportionately highlighted transcriptome/bioinformatics and machine learning, suggesting that mechanistic progress is increasingly driven by data-rich approaches. This transition is logical for macrophage biology: cell states are continuous, context-dependent, and difficult to capture with single markers. High-dimensional profiling can disentangle myeloid subsets, infer polarization trajectories, and link specific macrophage programs to biopsy phenotypes such as microvascular inflammation (40, 41). Machine learning, in turn, enables integration of multi-modal variables-histology, gene expression, clinical parameters-to predict rejection subtype, delayed graft function, or long-term decline, and to identify macrophage-associated feature sets with prognostic value (42, 43). The bibliometric signal therefore reflects a methodological “upgrade” that aligns with clinical needs: moving from mechanistic plausibility to predictive, patient-level stratification.

This study integrates WoSCC and PubMed to improve robustness: WoSCC enables comprehensive performance and knowledge-base analyses, while PubMed adds higher-resolution mechanistic and translational signals. Nevertheless, bibliometric findings are constrained by database coverage, keyword indexing variability, and citation-time bias. Additionally, network centrality indicates structural importance within literature, not biological causality. Future work should link bibliometric hotspots to evidence tiers to translate research attention into clinically actionable macrophage-directed strategies.

This study has several limitations that should be acknowledged. First, as a bibliometric analysis, the findings depend on the predefined search strategy, and although multiple synonymous terms were included, some relevant studies using alternative terminology may not have been fully captured, which is a common limitation in large-scale literature retrieval. Second, the analysis was based primarily on the Web of Science Core Collection and PubMed databases; therefore, database selection bias may exist, and relevant studies indexed in specialized or regional databases could have been omitted. Third, bibliometric indicators reflect structural and developmental patterns of the literature rather than direct clinical efficacy or biological causality. Thus, the identified hotspots should be interpreted as emerging research directions with translational potential rather than immediately validated clinical practices.

## 5 Conclusion

In summary, the field has evolved from descriptive macrophage infiltration toward mechanistic, cell-state–aware frameworks that connect microvascular inflammation and ABMR to chronic graft dysfunction, increasingly powered by transcriptomics, computational biology, and machine learning. These converging trajectories position macrophage programs as both mechanistic drivers and measurable biomarkers, offering a rational pathway toward precision monitoring and targeted immunomodulation in kidney transplantation.
